# Sex bias in systemic lupus erythematosus: a molecular insight

**DOI:** 10.1097/IN9.0000000000000004

**Published:** 2022-07-29

**Authors:** Moumita Bose, Caroline Jefferies

**Affiliations:** 1 Division of Rheumatology, Department of Medicine, Cedars-Sinai Medical Center, Los Angeles, CA, USA; 2 Research Division of Immunology, Department of Biomedical Sciences, Cedars-Sinai Medical Center, Los Angeles, CA, USA

**Keywords:** SLE, sex disparities, inflammation, estrogen, IFNα, X chromosome dosage, epigenetics, mitochondria

## Abstract

Acknowledging sex differences in immune response is particularly important when we consider the differences between men and women in the incidence of disease. For example, over 80% of autoimmune disease occurs in women, whereas men have a higher incidence of solid tumors compared to women. In general women have stronger innate and adaptive immune responses than men, explaining their ability to clear viral and bacterial infections faster, but also contributing to their increased susceptibility to autoimmune disease. The autoimmune disease systemic lupus erythematosus (SLE) is the archetypical sexually dimorphic disease, with 90% of patients being women. Various mechanisms have been suggested to account for the female prevalence of SLE, including sex hormones, X-linked genes, and epigenetic regulation of gene expression. Here, we will discuss how these mechanisms contribute to pathobiology of SLE and how type I interferons work with them to augment sex specific disease pathogenesis in SLE.

## 1. Introduction

Systemic lupus erythematosus (SLE) is a chronic, autoimmune, and multisystem disorder characterized by autoantibody production and dysregulated immune cell function. SLE is strongly sex-biased disease, affecting women nine times more frequently than men ^[1]^. Factors such as intrinsic differences in the immune system ^[2]^, sex hormones ^[3]^, sex differences in gene regulation, sex-dependent environmental factors ^[4]^ have been put forward to explain this sex bias. Sex hormones are an important contributor to sex bias in the immune response and in the incidence of disease as noted above ^[5–7]^. For example, estrogens in general are considered immunostimulatory for both innate and adaptive arms of the immune responses ^[8–10]^. On the other hand, androgens drive immunosuppressive or protective immune responses, leading to a lower prevalence of autoimmune diseases but a higher susceptibility to viral infection, one of the many mechanisms contributing to increased prevalence of COVID-19 in men ^[11]^. The changes in the hormonal milieu throughout the lifespan also impacts sex differences in disease. For example, prevalence in asthma and allergic diseases is higher in boys but, following puberty, the prevalence changes and adult females have a higher prevalence of asthma and allergic disease ^[12,13]^. Women also elicit a stronger innate immune response to viral infection, as evidenced by the recent COVID-19 outbreak. Differences in detection of and responses to viral nucleic acids may account for this. For example, the nucleic acid sensing Toll like receptors, *TLR7* and *TLR8* are encoded on the X chromosome and may escape X inactivation, leading to stronger expression in females than males. This in turn drives higher expression of the antiviral cytokines interferon (IFN)α and β, collectively called type I IFNs, in part explaining the stronger immune response in females compared to males in response to viral infection ^[14]^. In addition, plasmacytoid dendritic cells (pDCs) derived from women produce markedly more IFNα in response to TLR7 ligands than do pDCs from men ^[15]^. Estrogen receptor signaling, X chromosome dosage and enhanced levels of the transcription factor IRF5 (itself and estrogen regulated gene) have been shown to contribute to these enhanced IFN responses in female pDCs ^[16–18]^.

Although SLE is a complex, heterogeneous disease, there is a consensus that increased expression of type I IFN is an important driver of pathology. Approximately 50% of patients have increased expression of IFN-stimulated genes (ISG), which correlate positively with disease activity and severity ^[19,20]^. In SLE, uncontrolled nucleic acid sensing through TLRs or cytosolic nucleic acid sensors leads to aberrant production of IFNs and hyperactivation of the immune system and SLE flares ^[21–23]^. Indeed, IFNα levels in SLE patients correlate with increased expression of *TLR7*, promoting IFNα-driven gene expression ^[24]^. Moreover, immune cells from SLE female patients showed enhanced, monocyte antigen presentation and serum IgG level compared to male patients, driving a stronger humoral response and contributing to SLE vulnerability in female patients ^[25]^.

**Figure 1.**
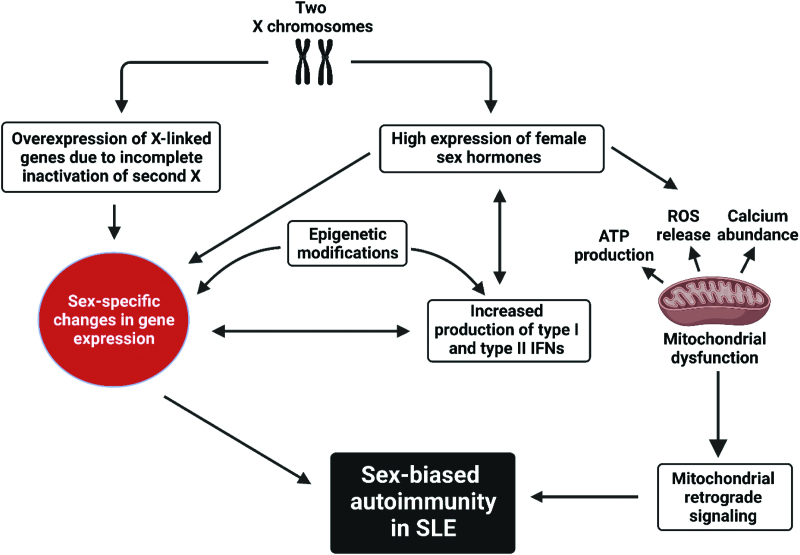
Overview of mechanisms that contribute to sex-bias in SLE. SLE: systemic lupus erythematosus.

Given the inherently sexually dimorphic nature of immune responses and particularly with respect to type I IFN, the female bias in SLE and other autoimmune diseases is it not surprising. This review will address likely mechanisms contributing to the sex bias in SLE, including mechanisms that contribute to altered immune reactivity and type I IFNs in SLE. The role of sex hormones, epigenetics and mitochondrial signaling in IFN production and SLE pathogenesis will be addressed (outlined in Figure 1), with emphasis on how these mechanisms contribute to disease pathology in SLE.

## 2. Influence of sex hormones and receptors in SLE

One of the strongest lines of evidence for a role for sex hormones in SLE comes from the observation that women with SLE experience disease exacerbations during pre-menstrual periods and pregnancy. In addition, clinical manifestations of the disease predominantly occur in women ages 20 to 50, the period of their lives when estrogen and progesterone levels are highest ^[26]^. In women, treatment with estrogen-containing medications increases susceptibility to developing SLE and, in SLE patients, increases risk of flares ^[27,28]^. Interestingly, supplementation of a transgender female with cross-gender hormones resulted in lupus nephritis, supporting a role for estrogen in driving SLE pathology ^[29]^. Animal studies also support a role for estrogen and female sex hormones in SLE. Ovariectomized lupus prone NZB/NZW F1 mice ameliorates disease, while estrogen supplementation in castrated male NZB/NZW F1 mice worsens the disease. In contrast, administration of androgen to female NZB/NZW F1 mice improves the SLE disease phenotype ^[30–33]^. Meanwhile, antagonizing estrogen using tamoxifen in this model reduces disease severity and lowers autoantibody levels ^[34]^. In addition, targeted B-cell deletion of *Esr1* reduces the production of autoantibodies and development of nephritis in NZB/NZW F1 mice ^[35]^.

However, studies in SLE patients have provided no real consensus regarding whether hormone imbalances exist in SLE ^[8]^. Similarly, use of oral contraceptives or hormonal replacement therapy have also been shown to be safe in SLE ^[36]^. So how might the link between changes in hormone levels during the lifetime of SLE patients and disease activity be explained? Estrogen mediates its effects by binding to estrogen receptors alpha and beta (ERα and ERβ), members of the steroid hormone nuclear receptor family that function as transcription factors that regulate gene expression. In their inactive form, they are in the cytosol but translocate to the nucleus and drive gene expression following estrogen binding. Receptors for progesterone (progesterone receptor) and testosterone (androgen receptor, AR) are also members of the steroid hormone nuclear receptor family of transcription factors. Interestingly, steady-state levels of ERα mRNA and protein have been reported to be significantly higher in immune cells from SLE patients compared with control ^[37,38]^. In addition, variation in a CAG repeat region in exon 1 of the AR, which reduces its signaling capacity (and hence immunomodulatory capacity) is associated with autoantibody production in SLE in both men and women ^[39,40]^. Murine models have also demonstrated ERα-deficiency in NZM2410 and MRL/lpr female (and not male mice) protects against kidney disease and prolongs survival ^[38,41,42]^.

Regarding how sex hormones affect the immune system, both AR and ER are expressed in a variety of immune cells, including lymphocytes and myeloid cells. Responses to estrogen can be both pro- or anti-inflammatory, depending on the cell type, organ microenvironment, or the relative expression of ERα and ERβ. Androgens and progesterone on the other hand are broadly immunosuppressive. Estrogen has broadly inflammatory effects on immune responses, triggering both direct and indirect expression of cytokines including IFNα and IFNγ, in addition to driving the upregulation of TLRs on macrophages in mice. In both mice and humans, estrogen and ER signaling is found to modulate the expression of immunostimulatory cytokines and exacerbates disease activity in lupus mouse model and in human patients ^[43]^. Previous studies indicated the influence of male and female sex hormones on immune cell functions in SLE ^[44]^. Particularly estrogen plays a pivotal role in functional modulation of T and B cells and their immune functions. It also regulates the expression of the transcription factor IRF5, which regulates the expression of inflammatory genes and IFNα and which has been identified as a risk factor for SLE in multiple GWAS studies ^[45]^. Sexually dimorphic expression of *IRF5* has been linked to higher IFNα in women ^[16]^. Another target of estrogen in SLE monocytes is the E3 ubiquitin ligase TRIM21 (also known as Ro52/SSA1), which regulates expression of the IRF family of transcription factors ^[37]^.

Studies have also indicated that genetic variations in ER genes might influence SLE susceptibility through the expression of cytokines, such as type I IFNs or IFNγ ^[38]^. Moreover, higher IFNα level corresponds to increased expression of ERα, indicating potential presence of a feedback loop between IFN and ERα signaling in SLE. Indeed, both IFN and ER signaling act together to further activate expression of IFN and estrogen responsive genes in SLE ^[38]^. Therefore, it is likely that the female sex hormone levels in women play a crucial role in the pathogenesis of SLE (and possibly in other autoimmune diseases) and sex-biased disease outcome. Of note, sex hormones play a crucial role in B-cell development and survival, with estrogen upregulating the expression of genes important for B-cell development and activation (CD22, SHP-1) and survival (Bcl-2) ^[46,47]^. Also, ERα and ERβ differentially regulate the maturation and selection of B cells and ERα but not ERβ can induce the development of autoimmunity through increased production of autoantibodies ^[8,48]^. For example, administration of the ERα agonist PPT to ovariectomized NZB/W mice increased autoantibody production and resulted in earlier disease onset compared with controls. However, treatment with the ERβ agonist DPN resulted in lower autoantibody production and reduced disease. Therefore, imbalance in signaling through the sex hormone receptors, either through increased expression of ERα or decreased signaling though AR, is sufficient to prime the immune system for autoreactivity through enhanced IFNα signaling or production.

## 3. X chromosome dosage and SLE

A number of lines of evidence support altered X chromosome inactivation (XCI) and gene-dose-effect as a driving factor for sex bias in SLE ^[49]^. For example, men with Klinefelter syndrome (47, XXY) have a 14-fold higher risk of SLE than do men with a single copy of the X chromosome (XY) ^[50]^. Furthermore, females with Turners’ syndrome [46, X, del(X)(q13)], where there is only one X chromosome, are less susceptible to get SLE, further supporting X-linked genes and gene-dosage in contributing to the female bias in SLE ^[51]^. DNA methylation of the X chromosome acts as an important epigenetic mechanism for maintaining gene-dose effect between sexes ^[52]^. In normal healthy females, one X chromosome is transcriptionally silenced by DNA methylation to prevent overexpression of X-linked genes ^[53]^. Failure to suppress X-linked genes on the inactive X chromosome contributes to sex bias in various autoimmune diseases including SLE ^[54]^. The long noncoding RNA (lncRNA) *XIST* is encoded by the X chromosome and is expressed in a female-specific manner in adult somatic cells ^[55,56]^. *XIST* is the major effector of XCI and deletions/mutation of *XIS*T reduces XCI ^[57]^. Recently, perturbations in *XIST* function have been associated with altered X inactivation and overexpression of X-linked genes in T cells from SLE patients ^[54]^. The X chromosome comprises several immune-related genes compared to the Y chromosome, such as *TLR7*, *TLR8*, *IL2RG*, and the transcription factor *FOXP3* ^[58]^. *IRAK1* is another X-linked gene, which is a key signaling component activated downstream of multiple TLRs ^[45]^. These and other immune-related X-linked genes are upregulated in SLE patients and demonstrate a female dominant expression pattern. For example, *CXCR3* (Xq13), *OGT* (Xq13), and *CD40LG* (Xq26) are overexpressed in CD4 ^+^ T cells of female SLE patients due to reduced methylation of their DNA ^[59]^. Similarly, *FOXP3* (Xp11) is also found to be significantly higher in female SLE CD8^+^ T cells although whether this is as a result of altered methylation is unknown ^[60]^. Specifically with respect to X chromatin inactivation, *TLR7* has been found to bypass X chromosome inactivation in pDCs, B cells and monocytes in SLE patients or men with Klinefelter syndrome, resulting in increased *TLR7* expression and TLR7 ligand responsiveness in B cells, driving them to differentiate into antibody secreting cells ^[61]^. Another TLR, *Tlr8* has been reported to increase IFNα production in 564Igi mice which have been crossed with *Tlr7/9*^*−/−*^ mice in females but not in males. Increased *Tlr8* expression is thought to be due to its location on the X chromosome, although whether it escapes XCI remains to be determined ^[62]^. Another functionally important gene that escapes X inactivation is the histone demethylase KDM6A ^[63]^. It regulates histone 3 lysine 27 trimethylation (H3K27me3) and in doing so regulates gene expression ^[64]^. Indeed, *KDM6A* was shown to be the most sexually dimorphic gene in CD4^+^ T cells from patients with multiple sclerosis and deletion of *Kdm6a* in mice was protective in experimental autoimmune encephalitis ^[65]^.

## 4. Epigenetic modifications and sexual dimorphism in SLE

### 4.1 DNA methylation

Among all epigenetic mechanisms, DNA methylation is most widely studied in SLE ^[66]^. Indeed, significantly lower expression of the DNA methylating enzymes, DNA methyl transferases (DNMTs), were evident in SLE patients compared to healthy control. Additional evidence for the role of methylation in SLE progression comes from studies using drugs that demethylate DNA. For example, 5-azacytidine, procainamide (DNMT inhibitor) and hydralazine (an ERK pathway signaling inhibitor) all induce SLE in rodents ^[67]^. Changes to DNA methylation also contribute to B cell–specific abnormalities in SLE. A recent study demonstrated that resting naïve B cells are epigenetically distinct in SLE in an African American SLE cohort with high disease activity ^[68]^. Another study demonstrated differences in B-cell DNA methylation (CpG) between African American and European American SLE patients in various development stages ^[69]^. Specifically, epigenetic abnormalities were evident in immature B cells from African American women with SLE, whereas abnormalities developed later during B-cell development in European American women with SLE. Enrichment of CpG sites was also reported in the IFN regulated genes (IRGs) in African American patients, suggesting ethnicity as an important factor regulating DNA methylation and potentially severity in SLE ^[70]^. Studies in SLE-prone mice also support a role for DNA methylation as being important for B-cell abnormalities in SLE. Specifically, the DNA methylases, Tet2, and Tet3, were shown to be important regulators of CD86 expression on self-reactive B cells, a mechanism that may contribute to female biased disease exacerbations. Deletion of *Tet2* and *Tet3* in B cells led to hyperactivation of B cells, autoantibody production, and lupus-like disease progression in mice ^[71]^. All these studies indicated a prominent role of immune cell–specific epigenetic mechanisms in SLE disease development.

### 4.2 Histone modifications

Various type of histone acetylation and methylation regulate chromatin accessibility to transcription factors and hence control gene expression. For example, acetylation of histones on lysine promotes more open chromatin and hence enhanced promoter accessibility ^[72]^. Methylation marks are more complex, with H3K4 trimethylation (me3) promoting recruitment of open chromatin remodeling complexes, such as the NURF complex. In contrast, H3K27me3 and H3K9me3 mediate transcriptional silencing ^[73]^. Sex differences in histone modifications have been associated with differences in learning and memory between males and females ^[74]^. Sexual dimorphism in the immune transcriptome has been suggested to be at the level of chromatin accessibility and regulated by type I IFN, suggesting potential sex differences in epigenetic remodeling may also underpin immune responses and potentially female-driven diseases such as SLE ^[75]^. Indeed, KDM6A, a histone demethylase specific for H3K27me3, is an X-linked gene, with interesting implications for female-driven disease ^[76]^. Indeed, both acetylation and methylation have been implicated in T-cell dysregulation and SLE pathogenesis by modulating immune-related gene expression. Hypomethylation of H3K27 positively corelated disease development whereas hypoacetylation of H3 was negatively associated with disease development ^[77]^. CD4^+^ T cells from SLE patients have been reported to have decreased histone acetylation and histone H3K9 methylation. The *IL17* gene cluster in SLE patient T cells was shown to have increased H3K18ac and reduced levels of H3K27me3 leading to uncontrolled expression of IL-17A ^[78]^. On the contrary, the *IL2* gene, which promotes immature T-cell differentiation into its effector phenotype, was found to be silent due to reduced histone acetylation and increased histone H3K9 methylation ^[79]^. Another study demonstrated that in T cells from SLE patients, increased expression of *IL10* (which correlates with the high disease activity) occurs as a result of modified histone acetylation and DNA methylation ^[80]^. All these observations suggest that altered gene expression due to inaccessibility of promoters by epigenetic modifications play a role in SLE development.

### 4.3 MicroRNAs and long noncoding RNA

Noncoding RNA (including miRNA and lncRNA) is found throughout the genome. However, startling differences between the X and Y chromosome exist with respect to miRNA content. Approximately 10% of all miRNAs (~120) are encoded by the X chromosome, whereas only 2 miRNAs are contained within the Y chromosome. A number of these X-linked miRNAs also regulate immune responses, suggesting a link between X-linked miRNAs and sexually dimorphic immune responses ^[81]^. Although a link with SLE has not been described to date, X-linked miRNAs have been found to be increased in patients with rheumatoid arthritis and increased between women and men with RA ^[82]^.

Dysregulated expression of miRNAs in SLE have been described by numerous groups ^[83–86]^. Many have multiple targets in multiple cell types, making their contribution to disease highly significant if their expression is dysregulated ^[86,87]^. Interestingly, expression of miRNA regulates epigenetic changes in gene expression. For example, miR148a plays a role in DNA hypomethylation of SLE CD4^+^ T cells by targeting DNMT1 and has higher expression in SLE patients than healthy individuals ^[88]^. In contrast, miR146a negatively regulates the IFNα pathway has lower expression in SLE patients ^[89]^. Estrogen regulation of microRNA expression has also been demonstrated with implications for SLE. For example, miR-125a is estrogen regulated, decreased in SLE and targets the inflammatory cytokine IL-16 and protects against pristane-induced lupus lung inflammation ^[90]^. Similarly, another estrogen regulated microRNA, miR-302d, targets the transcription factor IRF9 and regulates IFN-induced gene expression in SLE ^[91]^. Thus, the effects of sex hormones and X-linked expression on microRNA and lncRNA expression have a profound effect on gene expression with important implications for the sex bias of SLE.

## 5. Mitochondrial dysfunctions in SLE

Abnormal mitochondrial bioenergetics has been implicated in both animal models of SLE and human patients. For example, PBMCs from SLE patients were found to have significantly reduced mitochondrial complex-I activity compared to control ^[92]^, whereas chronic T-cell activation in SLE resulted into increased mitochondrial ATP production ^[93]^. T cells from SLE-prone mice displayed markedly increased energy production via activation of both OXPHOS and glycolysis compared to healthy control mice and that inhibition of metabolism using metformin or 2-deoxyglucose could reverse disease activity ^[94]^. These results suggest that alteration in mitochondrial function play a pivotal role in SLE development. Since SLE is a sex-biased disease, alteration of mitochondrial function may occur in a sex-biased manner, which ultimately affects disease phenotype ^[95,96]^. Recent studies indicated that mitochondrial functions such as calcium uptake, ROS generation, and quality control are influenced by sex ^[97]^. For example, ATP levels and mitochondrial content are higher in PBMCs from female blood compared to males ^[98]^. Recent evidence also indicates that estrogen may exert substantial effects on mitochondrial function which in turn may influence female-specific immune cell function and disease phenotype ^[99]^. For example, estrogen regulates (i) mitochondrial ATP and ROS production, (ii) mitochondrial calcium abundance, and (iii) activation of mitochondrial retrograde signaling ^[100,101]^, suggesting abnormal estrogen responses in SLE may have profound effect on mitochondrial function. Also, given the important role mitochondrial metabolites such as acetyl Co-A and α-ketoglutarate play in regulating histone modification, it will be interesting to study how these mitochondrial changes relate to epigenetic modifications and potentially contribute to sex bias in SLE.

## 6. Outlook

Understanding the molecular mechanisms regulating sexual dimorphism in immune responses is essential for the understanding of the female bias in SLE and how we might utilize this information to develop new therapies. Epigenetic studies in T and B cells from SLE patients have greatly enhanced our understanding of the role of these processes in regulating these cells in this disease. Identification of sex and cell type–specific epigenetic modifications, their downstream cellular pathways and how they impact IFN-driven immune responses in the context of SLE will be important in helping us understand the triggers contributing to disease susceptibility. IFN signaling may also be an important contributing factor for female-specific disease exacerbations by modulating cellular and mitochondrial pathways through a sex hormone dependent manner. Given the role that mitochondrial metabolites play in regulating epigenetic regulation of gene expression, it is intriguing to speculate that chronic IFN signaling may alter these pathways in specific cell types and contribute to disease pathology. Indeed, given the growing interest in immunometabolism in disease, understanding sex differences in how these pathways are regulated in specific immune cells will be important, particularly as drugs targeting metabolic pathways are currently being evaluated for various disease, including SLE. Similarly, environmental factors such as diet, drugs, or nutrition and the microbiome may also influence epigenetics and the expression of silenced genes in the inactive X chromosome ^[102]^. Further experimental evidence is required to understand the role of environmental factors in driving autoimmune pathology in SLE and whether these responses are sexually dimorphic.

## Conflicts of interest

The authors declare that they have no conflicts of interest.

## Funding

This work was supported by research funding from the National Institute of Allergy and Infectious Diseases (NIAID) of the National Institutes of Health under award number 1R01AI164504 (C.A. Jefferies). Support for this work has also come from the Center for Research in Women’s Health and Sex Differences (CREWHS) to Dr. Jefferies, Cedars Sinai Medical Center.

## References

[1] Danchenko N Satia JA Anthony MS. Epidemiology of systemic lupus erythematosus: a comparison of worldwide disease burden. Lupus. 2006;15:308–18. 16761508 10.1191/0961203306lu2305xx

[2] Klein SL Flanagan KL. Sex differences in immune responses. Nat Rev Immunol. 2016;16:626 8. 27546235 10.1038/nri.2016.90

[3] McMurray RW May W. Sex hormones and systemic lupus erythematosus: review and meta-analysis. Arthritis Rheum. 2003;48:2100–10. 12905462 10.1002/art.11105

[4] Parks RJ Ray G Bienvenu LA. Sex differences in SR Ca(2+) release in murine ventricular myocytes are regulated by the cAMP/PKA pathway. J Mol Cell Cardiol. 2014;75:162–73. 25066697 10.1016/j.yjmcc.2014.07.006

[5] Kaul A Gordon C Crow MK. Systemic lupus erythematosus. Nat Rev Dis Primers. 2016;2:16039. 27306639 10.1038/nrdp.2016.39

[6] Kim HI Lim H Moon A. Sex differences in cancer: epidemiology, genetics and therapy. Biomol Ther. 2018;26:335–42. 10.4062/biomolther.2018.103 PMC6029678 29949843

[7] Rubtsova K Marrack P Rubtsov AV. Sexual dimorphism in autoimmunity. J Clin Invest. 2015;125:2187–93. 25915581 10.1172/JCI78082 PMC4497744

[8] Moulton VR. Sex hormones in acquired immunity and autoimmune disease. Front Immunol. 2018;9:2279. 30337927 10.3389/fimmu.2018.02279 PMC6180207

[9] Ortona E Pierdominici M Rider V. Editorial: sex hormones and gender differences in immune responses. Front Immunol. 2019;10:1076. 31156632 10.3389/fimmu.2019.01076 PMC6530401

[10] Ngo ST Steyn FJ McCombe PA. Gender differences in autoimmune disease. Front Neuroendocrinol. 2014;35:347–69. 24793874 10.1016/j.yfrne.2014.04.004

[11] Gubbels Bupp MR Jorgensen TN. Androgen-induced immunosuppression. Front Immunol. 2018;9:794. 29755457 10.3389/fimmu.2018.00794 PMC5932344

[12] Shah R Newcomb DC. Sex bias in asthma prevalence and pathogenesis. Front Immunol. 2018;9:2997. 30619350 10.3389/fimmu.2018.02997 PMC6305471

[13] Barker DJ Eriksson JG Forsen T. Fetal origins of adult disease: strength of effects and biological basis. Int J Epidemiol. 2002;31:1235–9. 12540728 10.1093/ije/31.6.1235

[14] Bunders MJ Altfeld M. Implications of sex differences in immunity for SARS-CoV-2 pathogenesis and design of therapeutic interventions. Immunity. 2020;53:487–95. 32853545 10.1016/j.immuni.2020.08.003 PMC7430299

[15] Meier A Chang JJ Chan ES. Sex differences in the Toll-like receptor–mediated response of plasmacytoid dendritic cells to HIV-1. Nat Med. 2009;15:955–9. 19597505 10.1038/nm.2004 PMC2821111

[16] Griesbeck M Ziegler S Laffont S. Sex differences in plasmacytoid dendritic cell levels of IRF5 drive higher IFN-α production in women. J Immunol. 2015;195:5327–36. 26519527 10.4049/jimmunol.1501684 PMC4654231

[17] Laffont S Rouquié N Azar P. X-chromosome complement and estrogen receptor signaling independently contribute to the enhanced TLR7-mediated IFN-α production of plasmacytoid dendritic cells from women. J Immunol. 2014;193:5444–52. 25339659 10.4049/jimmunol.1303400

[18] Seillet C Laffont S Trémollières F. The TLR-mediated response of plasmacytoid dendritic cells is positively regulated by estradiol in vivo through cell-intrinsic estrogen receptor α signaling. Blood. 2012;119:454–64. 22096248 10.1182/blood-2011-08-371831

[19] Ronnblom L. The importance of the type I interferon system in autoimmunity. Clin Exp Rheumatol. 2016;34(4 Suppl 98):21–4. 27586799

[20] Crow MK Olferiev M Kirou KA. Type I interferons in autoimmune disease. Annu Rev Pathol. 2019;14:369–93. 30332560 10.1146/annurev-pathol-020117-043952

[21] Postal M Vivaldo JF Fernandez-Ruiz R. Type I interferon in the pathogenesis of systemic lupus erythematosus. Curr Opin Immunol. 2020;67:87–94. 33246136 10.1016/j.coi.2020.10.014 PMC8054829

[22] McWhirter SM Jefferies CA. Nucleic acid sensors as therapeutic targets for human disease. Immunity. 2020;53:78–97. 32668230 10.1016/j.immuni.2020.04.004

[23] Hagiwara AM Moore RE Wallace DJ. Regulation of cGAS-STING pathway-implications for systemic lupus erythematosus. J Rheumatol Immunol Res. 2021;2:173–84. 10.2478/rir-2021-0023 PMC9524788 36465073

[24] Celhar T Fairhurst AM. Toll-like receptors in systemic lupus erythematosus: potential for personalized treatment. Front Pharmacol. 2014;5:265. 25538618 10.3389/fphar.2014.00265 PMC4258990

[25] Singh RP Hahn BH Bischoff DS. Interferon genes are influenced by 17β-estradiol in SLE. Front Immunol. 2021;12:725325. 34733276 10.3389/fimmu.2021.725325 PMC8558410

[26] Tsokos GC. Systemic lupus erythematosus. N Engl J Med. 2011;365:2110–21. 22129255 10.1056/NEJMra1100359

[27] Tedeschi SK Bermas B Costenbader KH. Sexual disparities in the incidence and course of SLE and RA. Clin Immunol. 2013;149:211–8. 23578823 10.1016/j.clim.2013.03.003

[28] Lu LJ Wallace DJ Ishimori ML. Review: male systemic lupus erythematosus: a review of sex disparities in this disease. Lupus. 2010;19:119–29. 19946032 10.1177/0961203309350755 PMC7304291

[29] Hill BG Hodge B Misischia R. Lupus nephritis in a transgender woman on cross-sex hormone therapy: a case for the role of oestrogen in systemic lupus erythematosus. Lupus. 2020;29:1807–10. 32731807 10.1177/0961203320946372

[30] Michalski JP McCombs CC Roubinian JR. Effect of androgen therapy on survival and suppressor cell activity in aged NZB/NZW F1 hybrid mice. Clin Exp Immunol. 1983;52:229–33. 6222853 PMC1535569

[31] Roubinian JR Talal N Greenspan JS. Delayed androgen treatment prolongs survival in murine lupus. J Clin Invest. 1979;63:902–11. 447833 10.1172/JCI109390 PMC372031

[32] Roubinian JR Papoian R Talal N. Androgenic hormones modulate autoantibody responses and improve survival in murine lupus. J Clin Invest. 1977;59:1066–70. 864003 10.1172/JCI108729 PMC372318

[33] Roubinian JR Talal N Greenspan JS. Effect of castration and sex hormone treatment on survival, anti-nucleic acid antibodies, and glomerulonephritis in NZB/NZW F1 mice. J Exp Med. 1978;147:1568–83. 308087 10.1084/jem.147.6.1568 PMC2184317

[34] Sthoeger ZM Zinger H Mozes E. Beneficial effects of the anti-oestrogen tamoxifen on systemic lupus erythematosus of (NZBxNZW)F1 female mice are associated with specific reduction of IgG3 autoantibodies. Ann Rheum Dis. 2003;62:341–6. 12634234 10.1136/ard.62.4.341 PMC1754513

[35] Tabor DE Gould KA. Estrogen receptor alpha promotes lupus in (NZB×NZW)F1 mice in a B cell intrinsic manner. Clin Immunol. 2017;174:41–52. 27989899 10.1016/j.clim.2016.10.011 PMC5316311

[36] Rojas-Villarraga A Torres-Gonzalez JV Ruiz-Sternberg AM. Safety of hormonal replacement therapy and oral contraceptives in systemic lupus erythematosus: a systematic review and meta-analysis. PLoS One. 2014;9:e104303. 25137236 10.1371/journal.pone.0104303 PMC4138076

[37] Smith S Gabhann JN McCarthy E. Estrogen receptor α regulates tripartite motif-containing protein 21 expression, contributing to dysregulated cytokine production in systemic lupus erythematosus. Arthritis Rheumatol. 2014;66:163–72. 24449583 10.1002/art.38187

[38] Panchanathan R Shen H Zhang X. Mutually positive regulatory feedback loop between interferons and estrogen receptor-alpha in mice: implications for sex bias in autoimmunity. PLoS One. 2010;5:e10868. 20526365 10.1371/journal.pone.0010868 PMC2878324

[39] Olsen NJ Benko AL Kovacs WJ. Variation in the androgen receptor gene exon 1 CAG repeat correlates with manifestations of autoimmunity in women with lupus. J Endocr Connect. 2014;3:99–109. 10.1530/EC-14-0039 PMC4012646 24711544

[40] Tessnow A Olsen N Kovacs W. Expression of humoral autoimmunity is related to androgen receptor CAG repeat length in men with systemic lupus erythematosus. J Clin Immunol. 2011;31:567–73. 21445561 10.1007/s10875-011-9519-5 PMC3156391

[41] Svenson JL EuDaly J Ruiz P. Impact of estrogen receptor deficiency on disease expression in the NZM2410 lupus prone mouse. Clin Immunol. 2008;128:259–68. 18514033 10.1016/j.clim.2008.03.508 PMC4778964

[42] Perry DJ Yin Y Telarico T. Murine lupus susceptibility locus Sle1c2 mediates CD4^+^ T cell activation and maps to estrogen-related receptor gamma. J Immunol. 2012;189:793–803. 22711888 10.4049/jimmunol.1200411 PMC3392454

[43] Kassi E Moutsatsou P. Estrogen receptor signaling and its relationship to cytokines in systemic lupus erythematosus. J Biomed Biotechnol. 2010;2010:317452. 20617147 10.1155/2010/317452 PMC2896666

[44] Hui-Yuen JS Christiano AM Askanase A. Sex differences in genomics in lupus: girls with systemic lupus have high interferon gene expression while boys have high levels of tumour necrosis factor-related gene expression. Scand J Rheumatol. 2016;45:394–6. 26885894 10.3109/03009742.2015.1132760 PMC7920409

[45] Byrne JC Ni Gabhann J Lazzari E. Genetics of SLE: functional relevance for monocytes/macrophages in disease. Clin Dev Immunol. 2012;2012:582352. 23227085 10.1155/2012/582352 PMC3511832

[46] Grimaldi CM Cleary J Dagtas AS. Estrogen alters thresholds for B cell apoptosis and activation. J Clin Invest. 2002;109:1625–33. 12070310 10.1172/JCI14873 PMC151010

[47] Grimaldi CM Hill L Xu X. Hormonal modulation of B cell development and repertoire selection. Mol Immunol. 2005;42:811–20. 15829269 10.1016/j.molimm.2004.05.014

[48] Hill L Jeganathan V Chinnasamy P. Differential roles of estrogen receptors α and β in control of B-cell maturation and selection. Mol Med. 2011;17:211–20. 21107497 10.2119/molmed.2010.00172 PMC3060981

[49] Mousavi MJ Mahmoudi M Ghotloo S. Escape from X chromosome inactivation and female bias of autoimmune diseases. Mol Med. 2020;26:127. 33297945 10.1186/s10020-020-00256-1 PMC7727198

[50] Scofield RH Bruner GR Namjou B. Klinefelter’s syndrome (47,XXY) in male systemic lupus erythematosus patients: support for the notion of a gene-dose effect from the X chromosome. Arthritis Rheum. 2008;58:2511–7. 18668569 10.1002/art.23701 PMC2824898

[51] Cooney CM Bruner GR Aberle T. 46,X,del(X)(q13) Turner’s syndrome women with systemic lupus erythematosus in a pedigree multiplex for SLE. Genes Immun. 2009;10:478–81. 19458623 10.1038/gene.2009.37 PMC2722751

[52] Cotton AM Price EM Jones MJ. Landscape of DNA methylation on the X chromosome reflects CpG density, functional chromatin state and X-chromosome inactivation. Hum Mol Genet. 2015;24:1528–39. 25381334 10.1093/hmg/ddu564 PMC4381753

[53] Kalantry S. Recent advances in X-chromosome inactivation. J Cell Physiol. 2011;226:1714–8. 21344379 10.1002/jcp.22673 PMC3095210

[54] Syrett CM Paneru B Sandoval-Heglund D. Altered X-chromosome inactivation in T cells may promote sex-biased autoimmune diseases. JCI Insight. 2019;4:127. 10.1172/jci.insight.126751 PMC6483655 30944248

[55] Disteche CM Berletch JB. X-chromosome inactivation and escape. J Genet. 2015;94:591–9. 26690513 10.1007/s12041-015-0574-1 PMC4826282

[56] Youness A Miquel CH Guéry JC. Escape from X chromosome inactivation and the female predominance in autoimmune diseases. Int J Mol Sci. 2021;22:1114. 33498655 10.3390/ijms22031114 PMC7865432

[57] Kay GF Penny GD Patel D. Expression of Xist during mouse development suggests a role in the initiation of X chromosome inactivation. Cell. 1993;72:171–82. 8425217 10.1016/0092-8674(93)90658-d

[58] Bianchi I Lleo A Gershwin ME. The X chromosome and immune associated genes. J Autoimmun. 2012;38:J187–92. 22178198 10.1016/j.jaut.2011.11.012

[59] Hewagama A Gorelik G Patel D. Overexpression of X-linked genes in T cells from women with lupus. J Autoimmun. 2013;41:60–71. 23434382 10.1016/j.jaut.2012.12.006 PMC3622754

[60] Lu Q Wu A Tesmer L. Demethylation of CD40LG on the inactive X in T cells from women with lupus. J Immunol. 2007;179:6352–8. 17947713 10.4049/jimmunol.179.9.6352

[61] Souyris M Cenac C Azar P. TLR7 escapes X chromosome inactivation in immune cells. Sci Immunol. 2018;3:eaap8855. 29374079 10.1126/sciimmunol.aap8855

[62] Umiker BR Andersson S Fernandez L. Dosage of X-linked Toll-like receptor 8 determines gender differences in the development of systemic lupus erythematosus. Eur J Immunol. 2014;44:1503–16. 24500834 10.1002/eji.201344283 PMC4028042

[63] Greenfield A Carrel L Pennisi D. The UTX gene escapes X inactivation in mice and humans. Hum Mol Genet. 1998;7:737–42. 9499428 10.1093/hmg/7.4.737

[64] Gažová I Lengeling A Summers KM. Lysine demethylases KDM6A and UTY: The X and Y of histone demethylation. Mol Genet Metab. 2019;127:31–44. 31097364 10.1016/j.ymgme.2019.04.012

[65] Itoh Y Golden LC Itoh N. The X-linked histone demethylase Kdm6a in CD4^+^ T lymphocytes modulates autoimmunity. J Clin Invest. 2019;129:3852–63. 31403472 10.1172/JCI126250 PMC6715385

[66] Hurtado C Acevedo Sáenz LY Vásquez Trespalacios EM. DNA methylation changes on immune cells in systemic lupus erythematosus. Autoimmunity. 2020;53:114–21. 32019373 10.1080/08916934.2020.1722108 PMC8063264

[67] Jeffries MA Sawalha AH. Epigenetics in systemic lupus erythematosus: leading the way for specific therapeutic agents. Int J Clin Rheumtol. 2011;6:423–39. 22184503 10.2217/ijr.11.32 PMC3241218

[68] Scharer CD Blalock EL Mi T. Epigenetic programming underpins B cell dysfunction in human SLE. Nat Immunol. 2019;20:1071–82. 31263277 10.1038/s41590-019-0419-9 PMC6642679

[69] Breitbach ME Ramaker RC Roberts K. Population-specific patterns of epigenetic defects in the B cell lineage in patients with systemic lupus erythematosus. Arthritis Rheumatol. 2020;72:282–91. 31430064 10.1002/art.41083

[70] Adams DE Shao WH. Epigenetic alterations in immune cells of systemic lupus erythematosus and therapeutic implications. Cells. 2022;11:506. 35159315 10.3390/cells11030506 PMC8834103

[71] Tanaka S Ise W Inoue T. Tet2 and Tet3 in B cells are required to repress CD86 and prevent autoimmunity. Nat Immunol. 2020;21:950–61. 32572241 10.1038/s41590-020-0700-y

[72] Gujral P Mahajan V Lissaman AC. Histone acetylation and the role of histone deacetylases in normal cyclic endometrium. Reprod Biol Endocrinol. 2020;18:84. 32791974 10.1186/s12958-020-00637-5 PMC7425564

[73] Jambhekar A Dhall A Shi Y. Roles and regulation of histone methylation in animal development. Nat Rev Mol Cell Biol. 2019;20:625–41. 31267065 10.1038/s41580-019-0151-1 PMC6774358

[74] Keiser AA Wood MA. Examining the contribution of histone modification to sex differences in learning and memory. Learn Mem. 2019;26:318–31. 31416905 10.1101/lm.048850.118 PMC6699407

[75] Gal-Oz ST Maier B Yoshida H. ImmGen report: sexual dimorphism in the immune system transcriptome. Nat Commun. 2019;10:4295. 31541153 10.1038/s41467-019-12348-6 PMC6754408

[76] Hua C Chen J Li S. KDM6 demethylases and their roles in human cancers. Front Oncol. 2021;11:779918. 34950587 10.3389/fonc.2021.779918 PMC8688854

[77] Wu H Chen Y Zhu H. The pathogenic role of dysregulated epigenetic modifications in autoimmune diseases. Front Immunol. 2019;10:2305. 31611879 10.3389/fimmu.2019.02305 PMC6776919

[78] Hedrich CM. Epigenetics in SLE. Curr Rheumatol Rep. 2017;19:58. 28752494 10.1007/s11926-017-0685-1 PMC5532407

[79] Hedrich CM Rauen T Tsokos GC. cAMP-responsive element modulator (CREM)alpha protein signaling mediates epigenetic remodeling of the human interleukin-2 gene: implications in systemic lupus erythematosus. J Biol Chem. 2011;286:43429–36. 21976679 10.1074/jbc.M111.299339 PMC3234875

[80] Hedrich CM Rauen T Apostolidis SA. Stat3 promotes IL-10 expression in lupus T cells through trans-activation and chromatin remodeling. Proc Natl Acad Sci USA. 2014;111:13457–62. 25187566 10.1073/pnas.1408023111 PMC4169908

[81] Pinheiro I Dejager L Libert C. X-chromosome-located microRNAs in immunity: might they explain male/female differences? The X chromosome-genomic context may affect X-located miRNAs and downstream signaling, thereby contributing to the enhanced immune response of females. BioEssays. 2011;33:791–802. 21953569 10.1002/bies.201100047

[82] Khalifa O Pers YM Ferreira R. X-linked miRNAs Associated with gender differences in rheumatoid arthritis. Int J Mol Sci. 2016;17:1852. 10.3390/ijms17111852 PMC5133852 27834806

[83] Dai Y Huang YS Tang M. Microarray analysis of microRNA expression in peripheral blood cells of systemic lupus erythematosus patients. Lupus. 2007;16:939–46. 18042587 10.1177/0961203307084158

[84] Dai Y Sui W Lan H. Comprehensive analysis of microRNA expression patterns in renal biopsies of lupus nephritis patients. Rheumatol Int. 2009;29:749–54. 18998140 10.1007/s00296-008-0758-6

[85] Stagakis E Bertsias G Verginis P. Identification of novel microRNA signatures linked to human lupus disease activity and pathogenesis: miR-21 regulates aberrant T cell responses through regulation of PDCD4 expression. Ann Rheum Dis. 2011;70:1496–506. 21602271 10.1136/ard.2010.139857

[86] Zan H Tat C Casali P. MicroRNAs in lupus. Autoimmunity. 2014;47:272–85. 24826805 10.3109/08916934.2014.915955 PMC4239026

[87] Chi M Ma K Li Y. Immunological involvement of microRNAs in the key events of systemic lupus erythematosus. Front Immunol. 2021;12:699684. 34408748 10.3389/fimmu.2021.699684 PMC8365877

[88] Pan W Zhu S Yuan M. MicroRNA-21 and microRNA-148a contribute to DNA hypomethylation in lupus CD4^+^ T cells by directly and indirectly targeting DNA methyltransferase 1. J Immunol. 2010;184:6773–81. 20483747 10.4049/jimmunol.0904060

[89] Tang Y Luo X Cui H. MicroRNA-146A contributes to abnormal activation of the type I interferon pathway in human lupus by targeting the key signaling proteins. Arthritis Rheum. 2009;60:1065–75. 19333922 10.1002/art.24436

[90] Smith S Wu PW Seo JJ. IL-16/miR-125a axis controls neutrophil recruitment in pristane-induced lung inflammation. JCI Insight. 2018;3:e120798. 10.1172/jci.insight.120798 PMC6129123 30089723

[91] Smith S. MicroRNA-302d targets IRF9 to regulate the IFN-induced gene expression in SLE. J Autoimmun. 2017;79:105–11. 28318807 10.1016/j.jaut.2017.03.003

[92] Leishangthem BD Sharma A Bhatnagar A. Role of altered mitochondria functions in the pathogenesis of systemic lupus erythematosus. Lupus. 2016;25:272–81. 26385216 10.1177/0961203315605370

[93] Caza TN Talaber G Perl A. Metabolic regulation of organelle homeostasis in lupus T cells. Clin Immunol. 2012;144:200–13. 22836085 10.1016/j.clim.2012.07.001 PMC3423541

[94] Yin Y Choi SC Xu Z. Normalization of CD4^+^ T cell metabolism reverses lupus. Sci Transl Med. 2015;7:274ra18. 10.1126/scitranslmed.aaa0835 PMC5292723 25673763

[95] Ventura-Clapier R Moulin M Piquereau J. Mitochondria: a central target for sex differences in pathologies. Clin Sci. 2017;131:803–22. 10.1042/CS20160485 28424375

[96] Sultanova RF Schibalski R Yankelevich IA. Sex differences in renal mitochondrial function: a hormone-gous opportunity for research. Am J Physiol Renal Physiol. 2020;319:F1117–24. 33135479 10.1152/ajprenal.00320.2020 PMC7792688

[97] Parkash J Felty Q Roy D. Estrogen exerts a spatial and temporal influence on reactive oxygen species generation that precedes calcium uptake in high-capacity mitochondria: implications for rapid nongenomic signaling of cell growth. Biochemistry. 2006;45:2872–81. 16503642 10.1021/bi051855x

[98] Silaidos C Pilatus U Grewal R. Sex-associated differences in mitochondrial function in human peripheral blood mononuclear cells (PBMCs) and brain. Biol Sex Differ. 2018;9:34. 30045765 10.1186/s13293-018-0193-7 PMC6060503

[99] Klinge CM. Estrogenic control of mitochondrial function and biogenesis. J Cell Biochem. 2008;105:1342–51. 18846505 10.1002/jcb.21936 PMC2593138

[100] Hunt RJ Bateman JM. Mitochondrial retrograde signaling in the nervous system. FEBS Lett. 2018;592:663–78. 29086414 10.1002/1873-3468.12890

[101] Butow RA Avadhani NG. Mitochondrial signaling: the retrograde response. Mol Cell. 2004;14:1–15. 15068799 10.1016/s1097-2765(04)00179-0

[102] Pyfrom S Paneru B Knox JJ. The dynamic epigenetic regulation of the inactive X chromosome in healthy human B cells is dysregulated in lupus patients. Proc Natl Acad Sci USA. 2021;118:e2024624118. 34103397 10.1073/pnas.2024624118 PMC8214693

